# Preferential differences in vaccination decision-making for oneself or one’s child in The Netherlands: a discrete choice experiment

**DOI:** 10.1186/s12889-020-08844-w

**Published:** 2020-06-01

**Authors:** Joram Hoogink, Frederik Verelst, Roselinde Kessels, Albert Jan van Hoek, Aura Timen, Lander Willem, Philippe Beutels, Jacco Wallinga, G. Ardine de Wit

**Affiliations:** 1 Centre for Infectious Disease Control, National Institute for Public Health and the Environment, Bilthoven, The Netherlands; 2 Department of Biomedical Data Sciences, Leiden University Medical Centre, Leiden, The Netherlands; 3 Centre for Health Economics Research and Modelling Infectious Diseases, Vaccine and Infectious Disease Institute, University of Antwerp, Antwerp, Belgium; 4 Department of Data Analytics and Digitalization, Maastricht University, Maastricht, The Netherlands; 5 Department of Infectious Disease Epidemiology, London School of Hygiene & Tropical Medicine, London, England; 6 Athena Institute, Faculty of Earth and Life Sciences, VU University, Amsterdam, The Netherlands; 7 Centre for Nutrition, Prevention and Healthcare, National Institute for Public Health and the Environment, Bilthoven, The Netherlands; 8 Julius Centre Utrecht – University Medical Centre Utrecht, Utrecht University, Utrecht, The Netherlands

**Keywords:** Vaccination behaviour, Discrete choice experiment, Decision-making, Adult vaccination, Childhood vaccination

## Abstract

**Background:**

To optimize the focus of future public information campaigns in The Netherlands promoting the uptake of vaccines among adults and children, we quantified the contribution of several attributes to the vaccination decision.

**Method:**

We performed a discrete choice experiment (DCE) among Dutch adults including six attributes, i.e. vaccine effectiveness, vaccine-preventable burden of disease (specified in severity and frequency), accessibility of vaccination in terms of co-payment and prescription requirements, frequency of mild side-effects, population-level vaccination coverage and local vaccination coverage among family and friends. Participants answered the DCE from their own perspective (‘oneself’ group) or with regard to a vaccine decision for their youngest child (‘child’ group). The data was analysed by means of panel mixed logit models.

**Results:**

We included 1547 adult participants (825 ‘oneself’ and 722 ‘child’). Vaccine effectiveness was the most important attribute in the ‘oneself’ group, followed by burden of disease (relative importance (RI) 78%) and accessibility (RI 76%). In the ‘child’ group, burden of disease was most important, but tied closely with vaccine effectiveness (RI 97%). Of less importance was the risk of mild vaccine-related side-effects and both population and local vaccination coverage. Interestingly, participants were more willing to vaccinate when uptake among the population or family and friends was high, indicating that social influence and social norms plays a role.

**Conclusions:**

Vaccine effectiveness and disease severity are key attributes in vaccination decision-making for adults making a decision for themselves and for parents who decide for their children. Hence, public information campaigns for both adult and child vaccination should primarily focus on these two attributes. In addition, reinforcing social norms may be considered.

## Background

Vaccination is one of the major contributors to the global improvement of health and life expectancy [1, 2]. The success of vaccination programmes is dependent on a high uptake, which is currently threatened globally by an increase in vaccine hesitancy and refusal [3–6]. Since 2012, a decline in vaccine uptake has also been observed in The Netherlands, a country with high uptake rates historically [7]. To counter this decline, it is crucial to understand the importance of individual determinants of vaccine decision-making.

Determinants of vaccine decision-making have been studied thoroughly over the past years, revealing factors like (perceived) vaccine safety, risk perception, (perceived) vaccine effectiveness, and social norms and beliefs (for example religious or anthroposophical) [6, 8–11]. Multiple factors account for the recent decline in vaccine uptake, but two of the main drivers are lower vaccine confidence and risk perception, the latter referring to the perceived severity and susceptibility of vaccine-preventable diseases. Vaccine confidence is associated with the belief in vaccine effectiveness and safety [9, 12]. Risk perception is affected by the successes of vaccination programmes in the past, which have markedly decreased the incidence of many vaccine-preventable diseases. In this situation, individuals may increasingly become complacent to the threat and burden of these infectious diseases [13–15]. Closely linked to the concepts of risk perception and vaccine confidence is “freeriding” behaviour, i.e. relying on the vaccination of others and the effect of herd immunity to protect oneself [16], implying that high coverage leads to rational refusal of vaccination. This purely rational behaviour has been observed within experimental settings, where participants played so-called vaccination games [17]; however multiple observational studies have found evidence against this assumption [18, 19].

The Dutch vaccination programme consists of vaccinations that target different age and risk groups, including children, adolescents, pregnant women, clinical risk groups and elderly. It is likely that these different groups have a different inclination towards vaccination, based on different perspectives on individual risk and expectations with regard to future health. For example, an adult member of a risk group may have entirely different considerations than an adolescent, or a parent who has to make the decision about vaccinating a young child. Discrete choice experiments (DCE) have been used to study vaccine decision-making, both in The Netherlands and elsewhere [20–22]. These studies focussed either on adults taking decisions for themselves or on parents deciding for their children. None of these studies used the same DCE for both groups, hindering a direct comparison of preferences. Therefore, we have used a similar experimental design enabling a comparative study of vaccine decision-making between these two groups in The Netherlands.

## Methods

We employed a recently developed DCE design that was used in a study about vaccine decision-making in Flanders and South Africa [23, 24]. This choice design presented respondents with 10 choice sets of two hypothetical profiles of an unknown (general) vaccine against an unknown disease. For each choice set, respondents had to indicate their preferred vaccine profile. Besides respondents’ answers to the choice tasks, we gathered information on socio-economic factors as well as health literacy, in order to be able to explore the results for different groups. The survey was conducted in June–July 2018 among Dutch speaking adults in The Netherlands who are part of an online (registered) consumer panel. Recruitment of participants followed pre-defined sample quotas regarding background characteristics (Table 1). Sample quotas for gender and age were defined based on national statistics [25]. Generally, orthodox Protestants have a lower vaccine uptake due to religious beliefs [26–28]. To be able to capture their preferences, at least 10% of the respondents came from municipalities where more than 5% of the inhabitants voted for the orthodox Protestant political party (SGP) during the 2017 national elections [29]. Finally, for the purpose of being able to compare the decision with regard to a child to the decision for oneself we used a predefined sample quota of 50% inclusion of parents of at least one child below 18. Only one respondent per household could participate.

**Table 1:** Comparison of sample characteristics with pre-defined sample quota and population statistics

Characteristic	Adult group *N* = 825 (%)	Child group *N* = 722 (%)	Sample *N* = 1547 (%)	Dutch population (%)^a^	Predefined quota *N* = 1500 (%)
**Gender**					
Male	55.0	40.0	48.0	49.0	49.0
Female	45.0	60.0	52.0	51.0	51.0
**Age group**					
18–34	22.8	32.4	27.3	26.0	26.0
35–49	14.2	50.7	31.2	28.6	28.6
50–65	32.7	16.2	25.0	26.6	26.6
66–85	30.3	0.7	16.5	18.8	18.8
**Educational level**					
Low	21.9	12.9	17.7	31.4	
Medium	44.8	46.0	45.4	38.2	NA
High	33.2	41.1	36.9	28.9	
**Province**					
Drenthe	2.3	2.9	2.6	2.9	
Flevoland	1.7	1.7	1.7	2.4	
Friesland	4	4.4	4.2	3.8	
Gelderland	12.1	9.4	10.9	12.0	
Groningen	3.5	5.5	4.5	3.4	
Limburg	6.9	6.5	6.7	6.5	
Noord-Brabant	16.8	14.0	15.5	14.7	NA
Noord-Holland	13.8	17.9	15.7	16.5	
Overijssel	6.3	7.6	6.9	6.7	
Utrecht	7.5	6.6	6.9	7.5	
Zeeland	2.7	1.9	2.3	2.2	
Zuid-Holland	21.2	20.1	20.7	21.4	
Missing	1.1	1.4	1.2		
**Biblebelt**	17.1	9.8	13.7	3.0	10.0
**Cultural background**					
Dutch	90.4	88.2	89.4		
Western European	3.8	3.6	3.7		NA
Other	5.8	8.2	6.9		
**Household**					
Living alone	23.6	–	12.6		
Living alone with children	3.8	16.9	9.9		
Living together without children	40.8	–	21.8		NA
Living together with children	23.9	83.1	51.5		
Other	7.9	–	4.2		
**Health literacy (SBSQ) > 2**	91.1	90.7	90.9	NA	NA

^a^Source: Statistics Netherlands

We divided study participants in two groups, referred to as the ‘oneself’ and ‘child’ group. Respondents without children under 18 years of age could only be allocated to the ‘oneself’ group, remaining respondents were randomly allocated to either the ‘oneself’ or ‘child’ group. The ‘oneself’ group answered questions on vaccinating themselves, the ‘child’ group on vaccinating their youngest child. As an incentive for participation, respondents received credit rewards, transferable into coupons or air miles.

The questionnaire consisted of five parts involving (1) background characteristics of the respondent; (2) vaccine-related attitudes; (3) the DCE; (4) risk perception of infectious diseases and (5) health literacy. The full questionnaire is available in the Additional Material 1.

Background characteristics included: gender; age; 4-digit postal code; level of education; household composition; family size (number of children); age of youngest child; mother's country of birth; professional experience in the healthcare sector; previous exposure to severe diseases; eligibility for influenza vaccination (i.e. belonging to a risk group that qualifies for annual vaccination via the GP); past acceptance of influenza vaccination (if eligible); smoking status; beliefs that influence (attitude towards) vaccination decisions; religious background; and past acceptance of National Immunization Programme (NIP) vaccination for one’s children (if any).

The second part, surveying vaccine-related attitudes, contained 21 statements, each focussed on a particular aspect of the vaccination programme. These statements were adopted from a selection of previously conducted questionnaires on vaccine decision behaviour of parents as well as elderly [12, 22, 30]. All statements had to be answered on a 5-point Likert scale (mostly ranging from strongly disagree to strongly agree).

The third part, the DCE, assigned each respondent to 10 choice sets of two unlabelled vaccine profiles. To capture all main and interaction effects, we created a design of 50 choice sets, divided into five subsets of 10 choice sets, using a Bayesian D-optimal design [31]. The subsets were distributed randomly and evenly among the participants in both the ‘oneself’ and ‘child’ group. Each choice task was composed of six attributes, but only the levels of three of them differed between the two alternatives. These differences were made explicit by marking them in yellow. Respondents were, however, asked to take the levels of all attributes into account [31–34]. The attributes included were vaccine effectiveness, burden of disease for which the vaccine protects, vaccine-related side-effects (VRSE), accessibility of vaccination; population coverage and local coverage. Attributes were selected based on literature on DCEs in the context of vaccination [18–20, 35–38] and health economics in general [18–20, 32, 35–40], as well as qualitative studies. A focus group study (*n* = 16), pilot study (*n* = 41) and a soft launch with free-form feed-back (*n* = 184) in the original Flemish target population resulted in the final selection and finetuning of the six attributes. Attributes as burden of disease, vaccine effectiveness, VRSE and accessibility are utilized frequently in DCE studies in the field of vaccination. Vaccine coverage, both at the population and the local level, is included less often in vaccine DCEs, although its importance is well described in literature about behavioural change models [41]. For the full description of the survey design, we refer the interested reader to [24]. The attributes and levels of the DCE are shown in Table 2.

**Table 2:** DCE attributes and levels

Attribute	Level description
1. Vaccine effectiveness	a) Protects **50%** of vaccinated individuals
	b) Protects **90%** of vaccinated individuals
2. Burden of disease	a) The disease, against which the vaccine protects is **rare** and often **mild**: hospitalization is exceptional and the disease is not life-threatening
	b) The disease, against which the vaccine protects is **rare** and often **severe**: often with hospitalization and the disease is life-threatening
	c) The disease, against which the vaccine protects is **common** and often **mild**: hospitalization is exceptional and the disease is not life-threatening
	d) The disease, against which the vaccine protects is **common** and often **severe**: often with hospitalization and the disease is life-threatening
3. VRSE	a) Mild side-effects are **common** and severe side-effects are highly unlikely
	b) Mild side-effects **rarely occur** and severe side-effects are highly unlikely
4. Accessibility	a) The vaccine is **free of charge** and provided by the GP, well baby clinic or occupational physician
	b) The vaccine is **not reimbursed** and is only available with a prescription
5. Local coverage	a) **30%** of your acquaintances (friends and family) is already vaccinated
	b) **60%** of your acquaintances (friends and family) is already vaccinated
	c) **90%** of your acquaintances (friends and family) is already vaccinated
6. Population coverage	a) **30%** of the Dutch population is already vaccinated
	b) **60%** of the Dutch population is already vaccinated
	c) **90%** of the Dutch population is already vaccinated

The fourth part of the questionnaire assessed the participants’ perception of the relative severity of and susceptibility of themselves or their child for measles, influenza, a urinary tract infection and leukaemia [42]. Also, respondents had to indicate from which sources they received information or whom they would consult regarding vaccination decisions.

Finally, the respondents’ health literacy was assessed using Chew’s Set of Brief Screening Questions (SBSQ), which is a validated subjective measure of health literacy containing three items: ‘How confident are you filling out forms by yourself?’ (Confident with Forms), ‘How often do you have someone (like a family member, friend, hospital/clinic worker or caregiver) help you read hospital materials?’ (Help Read), and ‘How often do you have problems learning about your medical condition because of difficulty understanding written information?’ (Problems Reading) [43–45]. Responses were scored on a 5-point Likert scale ranging from 0 (always or not certain at all) to 4 (never or completely certain). A total score, taken as the mean over the three items, with a value of ≤2 indicates inadequate health literacy [43–45].

Note that the entire questionnaire was identical for the ‘oneself’ and ‘child’ group except for the framing of the subject of interest with respect to the vaccination decision (oneself or one’s child). As indicated, the design of our Dutch DCE was identical to the design of the South African DCE [23], which was an improved version of the design used in Flanders [24]. The design was explicitly developed to be used for cross-country comparisons. The additional background questions of our study were, however, tailored to the Dutch situation, as was the addition of health literacy items. Our changes to the original questionnaires were reviewed and accepted by the Ethical Committee of the Antwerp University Hospital (UZA, Belgium), granting ethical clearance to perform this study (Reference number: 15/2/12).

The relative importance of the attributes and the relative utility values attached to the attribute levels were obtained by a Panel Mixed Logit (PML) model using Hierarchical Bayes estimation. To accommodate for unobserved preference heterogeneity of the respondents, we assumed normally distributed preference parameters without correlation between attributes. We ran 10,000 Bayesian iterations, and used the last 5000 for estimation. The total utility of a vaccination alternative is the sum of the attributes’ main and interaction effect estimates. Overall significance of the attributes was computed by likelihood ratio (LR) tests and relative importance of each attribute by the normalized logworth statistic (*−log*_10_ (*p*-value of the LR-test)). Based on the finding that both coverage attributes depict a linear trend, we used linear coding for these attributes. Therefore, the estimates for these attributes represent the marginal change in utility when vaccination coverage increases by 10%. The DCE results were analysed using the Choice Modelling platform of JMP Pro 14 [46].

We estimated the a priori PML model consisting of all main attribute effects and all two-way interactions between an attribute and ‘vaccine effectiveness’, ‘VRSE’ and ‘accessibility’, for both the ‘oneself’ and ‘child’ group. We explored structural differences in observed preference heterogeneity among groups of respondents by estimating all two-way interactions between the vaccine attributes and the background characteristics, attitudes towards vaccination, risk perception and health literacy questions in separate models. Next, we studied all individually significant two-way interactions in a joint model, where we iteratively dropped all insignificant covariates until we obtained a joint model consisting of only significant terms. As final step we removed interaction terms with a relative importance below a normalized logworth value of 4.

## Results

The final dataset consisted of 1547 respondents, divided into the ‘oneself’ group (*n* = 825) and the ‘child’ group (*n* = 722). The study sample was representative of the general Dutch population with respect to the background characteristics gender, age and province. There was an underrepresentation of individuals with lower educational attainment. The ‘oneself’ and the ‘child’ groups differed slightly with regard to age and gender (see Table 1) because assignment to the ‘child’ group was conditional on being a parent of a child below 18 years, which is closely tied to the age of the parent. With regard to health literacy, 90*.*9% of the total sample had a health literacy mean score above the threshold set by Chen et al. (*>* 2), indicating that the vast majority of the respondents were health literate (Table 1).

With regard to all respondents, both in the ‘oneself’ and the child group, with children below 18 years old (*N* = 855), 85.5% report that they fully vaccinated their child (ren) following the NIP guidelines, 6.5% did not vaccinate, 5.7% vaccinated their child(ren) partially and the remaining indicated ‘n/a’ (2.3%). Based on dichotomized 5-point Likert responses on three statements (‘Vaccinating myself/my child against infectious diseases is wise’, ‘Vaccinating myself/my child against infectious disease is important’, ‘Vaccinating myself/my child against infectious diseases is necessary), at least 80% of the parents in the ‘child’ group indicated a positive attitude towards vaccination for each statement separately, whereas this held for 75% in the ‘oneself’ group. Table 3 summarizes the scores on the attitudinal items for the two groups, where answers were grouped in (completely) disagree, neutral and (completely) agree, in terms of percentages of respondents who agree (in most cases truly positive) and who provided a neutral score. For both the ‘oneself’ and the ‘child’ group, around 5% were truly negative about all statements. The respondents in the ‘child’ group were more positive overall regarding the attitudinal questions compared to the respondents in the ‘oneself’ group. Strikingly, a large proportion provided a neutral score on average over all items: 27% in the ‘oneself’ group and 22.3% in the ‘child’ group. With one exeption, these background variables did not provide relevant associations that could explain differences in decisions made in the DCE. The exception was in case of the ‘oneself’ group, where the item “If I do not have myself vaccinated, there is a good chance that I experience an infectious disease against which vaccination is done within the NIP” (risk perception), was important for explaining preference heterogeneity.

**Table 3:** Distribution of answers on attitudinal items by respondent in oneself and child group

Statement Adult	Statement Child	Answer range	% > 3 Oneself	% neutral Oneself	% > 3 Child	% neutral Child
I find vaccinating myself against infectious disease	Vaccinating my child following the NIP is	Very unwise (1) – very wise (5)	79.2	14.8	81.9	12.6
I find vaccinating myself against infectious disease	Vaccinating my child following the NIP is	Very unimportant (1) - very important (5)	78.3	16.4	83.0	12.6
I find vaccinating myself against infectious disease	Vaccinating my child following the NIP is	Very unnecessary (1) - very necessary (5)	72.5	21.1	81.4	13.0
People who are important to me, find that I have to vaccinate myself	People who are important to me, think that I have to vaccinate my child	Totally disagree (1) - totally agree (5)	46.5	39.3	56.9	33.4
People who are important to me vaccinate themselves	People who are important to me vaccinate their child (ren)	Totally disagree (1) - totally agree (5)	57.3	33.6	71.9	22.2
People who are important to me appreciate if I vaccinate myself	People who are important to me appreciate if I vaccinate my child	Totally disagree (1) - totally agree (5)	51.4	40.1	59.8	33.2
If I do not have myself vaccinated, there is a good chance that I experience an infectious disease against which vaccination is done within the NIP	If I do not have my child vaccinated, there is a good chance that he / she will develop an infectious disease against which vaccination is done within the NIP	Totally disagree (1) - totally agree (5)	43.4	40.0	56.6	30.6
The NIP is good for the protection of my own health	The NIP is good for the protection of the health of my child	Totally disagree (1) - totally agree (5)	72.0	21.2	79.5	15.0
I find it important that vaccinating myself contributes to the protection of others	I find it important that vaccinating my child contributes to the protection of others	Totally disagree (1) - totally agree (5)	72.8	21.2	75.3	20.1
The infectious diseases against which vaccination is carried out can be very severe	The infectious diseases against which vaccination is carried out can be very severe	Totally disagree (1) - totally agree (5)	79.5	17.0	82.8	14.4
The adverse side-effects of the vaccinations within the NIP can be very severe	The adverse side-effects of the vaccinations within the NIP can be very severe	Totally disagree (1) - totally agree (5)	26.3	48.2	23.4	43.4
Vaccinating myself is not something I have to think hard about	Vaccinating my child is not something I have to think hard about	Totally disagree (1) - totally agree (5)	72.8	19.2	75.6	15.8
Vaccinating myself is self-evident	Vaccinating my child is self-evident	Totally disagree (1) - totally agree (5)	67.0	20.8	75.9	15.5
If I have to decide now, I would vaccinate myself	If I have to decide now, I would vaccinate my child following the NIP	Totally disagree (1) - totally agree (5)	70.4	20.0	79.1	14.0
If I had to make an extra appointment with my healthcare provider for vaccination, that would be a reason not to get vaccinated	If I had to make an extra appointment with my healthcare provider for vaccination, that would be a reason not to have my child (ren) vaccinated	Totally disagree (1) - totally agree (5)	12.4	20.6	18.1	18.1
I trust the information about vaccinations that I receive from the healthcare provider	I trust the information about vaccinations that I receive from the healthcare provider	Totally disagree (1) - totally agree (5)	72.4	20.5	72.8	19.9
I trust the information about vaccinations that I receive from the government	I trust the information about vaccinations that I receive from the government	Totally disagree (1) - totally agree (5)	67.9	22.5	70.4	19.5
It’s everyone’s responsibility to get vaccinated	It’s the responsibility of every parent to vaccinate their child (ren)	Totally disagree (1) - totally agree (5)	69.2	21.1	69.7	20.8
I find it bad when others do not vaccinate	I find it bad when other parents do not vaccinate their child (ren)	Totally disagree (1) - totally agree (5)	59.9	27.2	59.8	27.8
Experiencing infectious diseases contributes to a positive mental and physical development	Experiencing infectious diseases contributes to a positive mental and physical development of my child	Totally disagree (1) - totally agree (5)	30.1	40.1	33.4	35.2
Experiencing infectious diseases leads to a better and life-long protection compared to vaccination	Experiencing infectious diseases leads to a better and life-long protection compared to vaccination	Totally disagree (1) - totally agree (5)	29.8	42.2	34.1	30.7

Wording of statements differs between the ‘oneself’ and ‘child’ groups. Items are scored on a 5-point Likert scale, with specific wording (see Answer range). Per group the percentages of respondents who provided a score larger than 3 and a neutral score of 3 are given

### ‘Oneself’ model

**Fig. 1:**
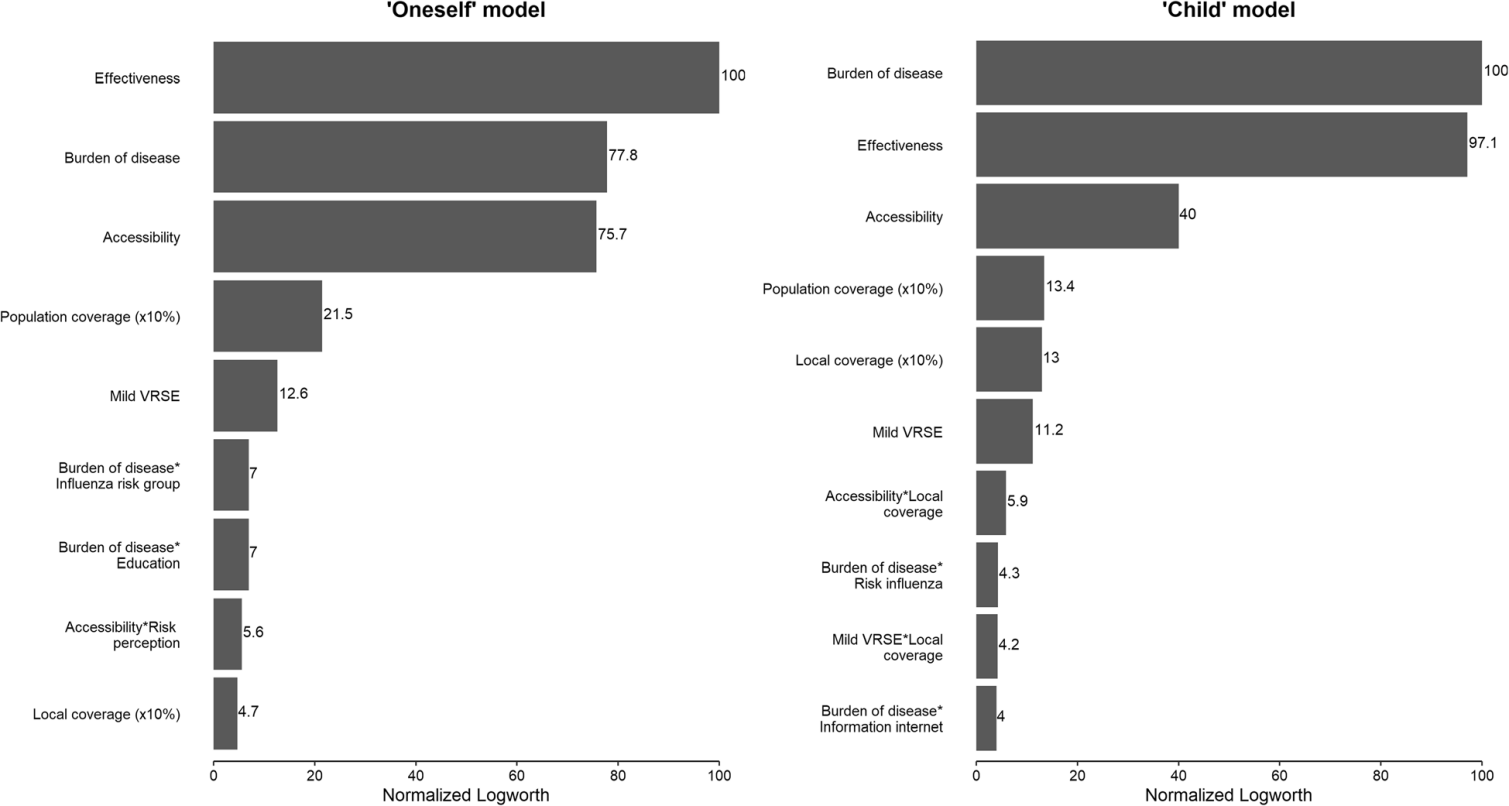
Importance of all statistically significant main and interaction effects (*p* < 0.05) relative to the most important attribute ‘vaccine effectiveness’ (‘oneself’ model) and ‘burden of disease’ (‘child’ model)

For respondents in the ‘oneself’ group, vaccine effectiveness was the most important attribute in their vaccine decision, followed by burden of disease and accessibility, which were practically equally important (relative importance of 78 and 76% compared to vaccine effectiveness – Fig. 1). Far less influential were population coverage and mild VRSE, with relative importance of 22 and 13%. Local coverage (5%) was ranked last, after several covariate interactions indicating preference heterogeneity. For both population and local coverage, respondents preferred a vaccine that has a high uptake already. In Table 4, the estimates for the ‘oneself’ model are displayed, representing marginal utilities assigned to the different attribute levels. Noteworthy is that for burden of disease, the mild levels do not significantly differ (− 0.764 vs. -0.753), indicating no strong preference for one over the other. For all estimates hold that a more positive marginal utility translates to a more preferable vaccine profile. As described, burden of disease is expressed in terms of severity and susceptibility. The estimates show that severity is assigned more weight, indicating that a vaccine is preferred against a rare but severe disease over one that protects against a more frequent but mild disease.

**Table 4:** Panel mixed logit model estimates of the ‘oneself’ model: mean and standard deviation (std dev) and significance of the attribute effects obtained from likelihood ratio (LR) tests with specified number of degrees of freedom (DF)

Term	Mean estimate (std dev; subject std dev)	95% CI	Marginal utilitiy	LR Chi-square	DF	*P*-value
**Vaccine effectiveness**						
50%	−0.755 (0.039; 0.323)	[−0.833; − 0.682]	−0.7551	383.159	1	<.0001
90%	0.755 (0.036; 0.293)	[0.685; 0.825]	0.7551			
**Burden of disease**						
Rare&mild	−0.764 (0.080; 0.225)	[− 0.953; − 0.632]	−0.7642	308.482	3	<.0001
Common&mild	−0.753 (0.079; 0.204)	[− 0.900; − 0.595]	−0.7533			
Rare&severe	0.351 (0.073; 0.144)	[0.201; 0.497]	0.3511			
Common&severe	1.166 (0.076; 0.180)	[1.018; 1.315]	1.1664			
**Mild VRSE**						
Common	−0.210 (0.027; 0.102)	[− 0.264; − 0.159]	−0.2103	44.657	1	<.0001
Rare	0.210 (0.025; 0.106)	[0.160; 0.260]	0.2103			
**Accessibility**						
Co-payment prescription	−0.608 (0.041; 0.240)	[− 0.691; − 0.533]	−0.6085	288.679	1	<.0001
Free accessible	0.608 (0.044; 0.242)	[0.522; 0.695]	0.6085			
**Local coverage (×10%)**	0.054 (0.012; 0.122)	[0.031; 0.078]	0.0538	15.09	1	0.0001
**Global coverage (× 10%)**	0.125 (0.013; 0.160)	[0.101; 0.153]	0.1247	79.101	1	<.0001
Burden of disease*Educational level						
Rare&mild*Low	0.503 (0.134; 0.247)	[0.254; 0.789]	−0.2617	38.004	6	<.0001
Rare&mild*Mid	−0.099 (0.108; 0.283)	[− 0.291; 0.169]	−0.8635			
Rare&mild*High	−0.403 (0.082; 0.182)	[−0.563; − 0.243]	−1.1675			
Common&mild*Low	0.196 (0.132; 0.275)	[−0.046; 0.451]	−0.5572			
Common&mild*Mid	−0.005 (0.098; 0.246)	[−0.195; 0.174]	−0.7586			
Common&mild*High	−0.191 (0.094; 0.242)	[−0.375; − 0.006]	−0.9441			
Rare&severe*Low	−0.174 (0.092; 0.180)	[−0.365; − 0.014]	0.1774			
Rare&severe*Mid	0.182 (0.071; 0.134)	[0.053; 0.316]	0.5332			
Rare&severe*High	−0.008 (0.091; 0.161)	[−0.186; 0.170]	0.3427			
Common&severe*Low	−0.525 (0.094; 0.198)	[− 0.708; − 0.341]	0.6415			
Common&severe*Mid	−0.078 (0.079; 0.167)	[−0.232; 0.077]	1.0888			
Common&severe*High	0.602 (0.093; 0.200)	[0.419; 0.785]	1.7688			
Burden of disease*Being target group for influenza vaccination						
Rare&mild*Yes	0.216 (0.067; 0.190)	[0.093; 0.359]	−0.5482	30.472	3	<.0001
Common&mild*Yes	0.179 (0.072; 0.203)	[0.034; 0.313]	−0.5742			
Rare&severe*Yes	−0.087 (0.063; 0.133)	[−0.217; 0.040]	0.2638			
Common&severe*Yes	−0.308 (0.063; 0.149)	[−0.431; −0.184]	0.8586			
Rare&mild*No	−0.216 (0.067; 0.145)	[−0.348; −0.084]	−0.9802			
Common&mild*No	−0.179 (0.074; 0.187)	[−0.323; −0.035]	−0.9324			
Rare&severe*No	0.087 (0.062; 0.116)	[−0.034; 0.209]	0.4384			
Common&severe*No	0.308 (0.060; 0.132)	[0.191; 0.425]	1.4742			
Accessibility*Risk perception Low vs High						
Co-payment*Low	0.158 (0.037; 0.256)	[0.091; 0.233]	−0.4500	18.311	1	<.0001
Co-payment*High	−0.158 (0.037; 0.272)	[−0.231; −0.085]	−0.7669			
Free*Low	−0.158 (0.043; 0.228)	[−0.242; −0.074]	0.4500			
Free*High	0.158 (0.037; 0.253)	[0.087; 0.230]	0.7669			

Note: Mean estimates corresponding to the last level of an attribute were calculated as minus the sum of the estimates for the other levels of the attribute

**Fig. 2:**
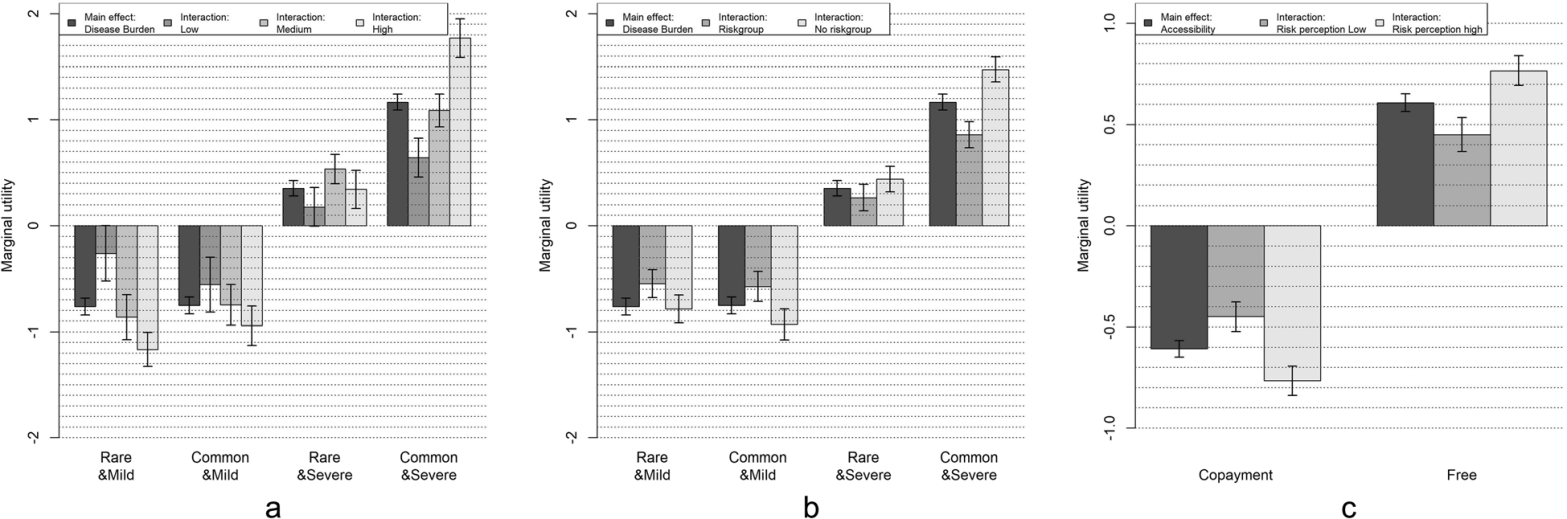
Marginal utilities for significant covariate interaction terms in the ‘oneself’ model. **a** Covariate interaction between burden of disease and educational level. **b** Covariate interaction between burden of disease and influenza riskgroup. **c** Covariate interaction between accessibility and disease risk perception

With regard to preference heterogeneity evidenced by significant covariate interactions (Fig. 2 and Table 4), we found that respondents with a lower level of education attached less importance to the disease burden compared to those with a higher educational level. In the same line, respondents belonging to the target group for annual influenza vaccination (i.e., being over 60 years of age and/or having one or more chronic diseases) were less sensitive towards the disease burden compared to those who are not eligible. Another significant interaction involved the response to the statement ‘The infectious diseases against which vaccination is carried out can be very severe (risk perception)’ (79.5% of the subsample in the ‘oneself’ group). Respondents who did agree with this statement had stronger preferences for accessibility compared to those who did not agree.

### ‘Child’ model

The best fitting ‘child’ model differed slightly from the ‘oneself’ model. Vaccine effectiveness and burden of disease were considered equally important in the ‘child’ model (Fig. 1). In addition, accessibility and population coverage were of lower importance in the ‘child’ model than in the ‘oneself’ model, but local coverage was of higher importance. Table 5 presents marginal utility estimates, where, like in the ‘oneself’ model, the least attractive levels of vaccine effectiveness, burden of disease and accessibility all had a substantial negative effect on total utility, and vice versa for the most attractive levels. As in the ‘oneself’ model, the vaccines protecting against the more severe diseases are preferred over the mild variants. In contrast though, here a vaccine against a frequently occurring mild disease is significantly preferred over the rare mild variant. A 10% increase in population coverage led to a marginal utility increase of 0.112, implying that vaccines with a higher global coverage were preferred. In case of local coverage, the marginal utility was dependent on accessibility and mild VRSE, due to significant interaction effects. In both cases, we find a positive addition to the less preferable levels of VRSE (more VRSE) and accessibility (need for co-payment) when local coverage is high.

**Table 5:** Panel mixed logit model estimates of the ‘child’ model: mean and standard deviation (std dev) and significance of the attribute effects obtained from likelihood ratio (LR) tests with specified number of degrees of freedom (DF)

Term	Mean estimate (std dev; subject std dev)	95% CI	Marginal utility	LR Chi-square	DF	*P*-value
**Vaccine effectiveness**						
50%	−0.970 (0.047; 0.366)	[−1.070; −0.888]	−0.9701	382.034	1	<.0001
90%	0.970 (0.056; 0.380)	[0.861; 1.079]	0.9701			
**Burden of disease**						
Rare&mild	−1.507 (0.154; 0.377)	[−1.811; −1.198]	−1.5066	405.516	3	<.0001
Common&mild	−0.969 (0.105; 0.311)	[−1.179; −0.761]	−0.9686			
Rare&severe	0.683 (0.099; 0.175)	[0.458; 0.842]	0.6831			
Common&severe	1.792 (0.108; 0.506)	[1.580; 2.004]	1.7921			
**Mild VRSE**						
Common	−0.518 (0.055; 0.116)	[−0.637; −0.417]	−0.5175	40.569	1	<.0001
Rare	0.518 (0.071; 0.120)	[0.379; 0.656]	0.5175			
**Accessibility**						
Co-payment prescription	−0.971 (0.059; 0.177)	[−1.098; −0.870]	−0.9713	154.293	1	<.0001
Free accessible	0.971 (0.092; 0.172)	[0.791; 1.152]	0.9713			
**Local coverage (× 10%)**	0.115 (0.016; 0.172)	[0.084; 0.146]	0.1147	47.791	1	<.0001
**Global coverage (×10%)**	0.112 (0.013; 0.141)	[0.086; 0.139]	0.1119	49.112	1	<.0001
Mild VRSE*Local coverage						
Common*Local coverage	0.041 (0.010; 0.051)	[0.024; 0.061]	0.0412	13.527	1	0.0002
Rare*Local coverage	−0.041 (0.012; 0.052)	[−0.064; −0.018]	−0.0412			
Accessibility*Local coverage						
Co-payment*local	0.044 (0.009; 0.080)	[0.028; 0.063]	0.0444	20.147	1	<.0001
Free*local	−0.044 (0.014; 0.085)	[−0.072; −0.017]	−0.0444			
Burden of disease*Risk of flu next 12 month (Low vs. high)						
Rare&mild*High	−0.260 (0.112; 0.378)	[−0.471; −0.047]	−1.7664	19.799	3	0.0002
Common&mild*High	−0.155 (0.093; 0.303)	[−0.371; 0.007]	−1.1238			
Rare&severe*High	−0.003 (0.086; 0.156)	[−0.186; 0.145]	0.6803			
Common&severe*High	0.418 (0.094; 0.367)	[0.234; 0.602]	2.2098			
Rare&mild*Low	0.260 (0.104; 0.489)	[0.057; 0.463]	−1.2469			
Common&mild*Low	0.155 (0.093; 0.245)	[−0.028; 0.338]	−0.8134			
Rare&severe*Low	0.003 (0.098; 0.216)	[−0.189; 0.194]	0.6859			
Common&severe*Low	−0.418 (0.094; 0.601)	[−0.602; −0.233]	1.3744			
Burden of disease*Information via internet (other than social media)						
Rare&mild*No	0.427 (0.099; 0.475)	[0.251; 0.625]	−1.0800	18.541	3	0.0003
Common&mild*No	0.007 (0.083; 0.256)	[−0.143; 0.184]	−0.9611			
Rare&severe*No	−0.048 (0.075; 0.183)	[−0.182; 0.113]	0.6346			
Common&severe*No	−0.386 (0.096; 0.416)	[−0.575; −0.196]	1.4066			
Rare&mild*Yes	−0.427 (0.111; 0.296)	[−0.645; −0.208]	−1.9332			
Common&mild*Yes	−0.007 (0.082; 0.189)	[−0.167; 0.152]	−0.9760			
Rare&severe*Yes	0.048 (0.086; 0.204)	[−0.120; 0.217]	0.7316			
Common&severe*Yes	0.386 (0.094; 0.306)	[0.202; 0.569]	2.1777			

Note: Mean estimates corresponding to the last level of an attribute were calculated as minus the sum of the estimates for the other levels of the attribute

**Fig. 3:**
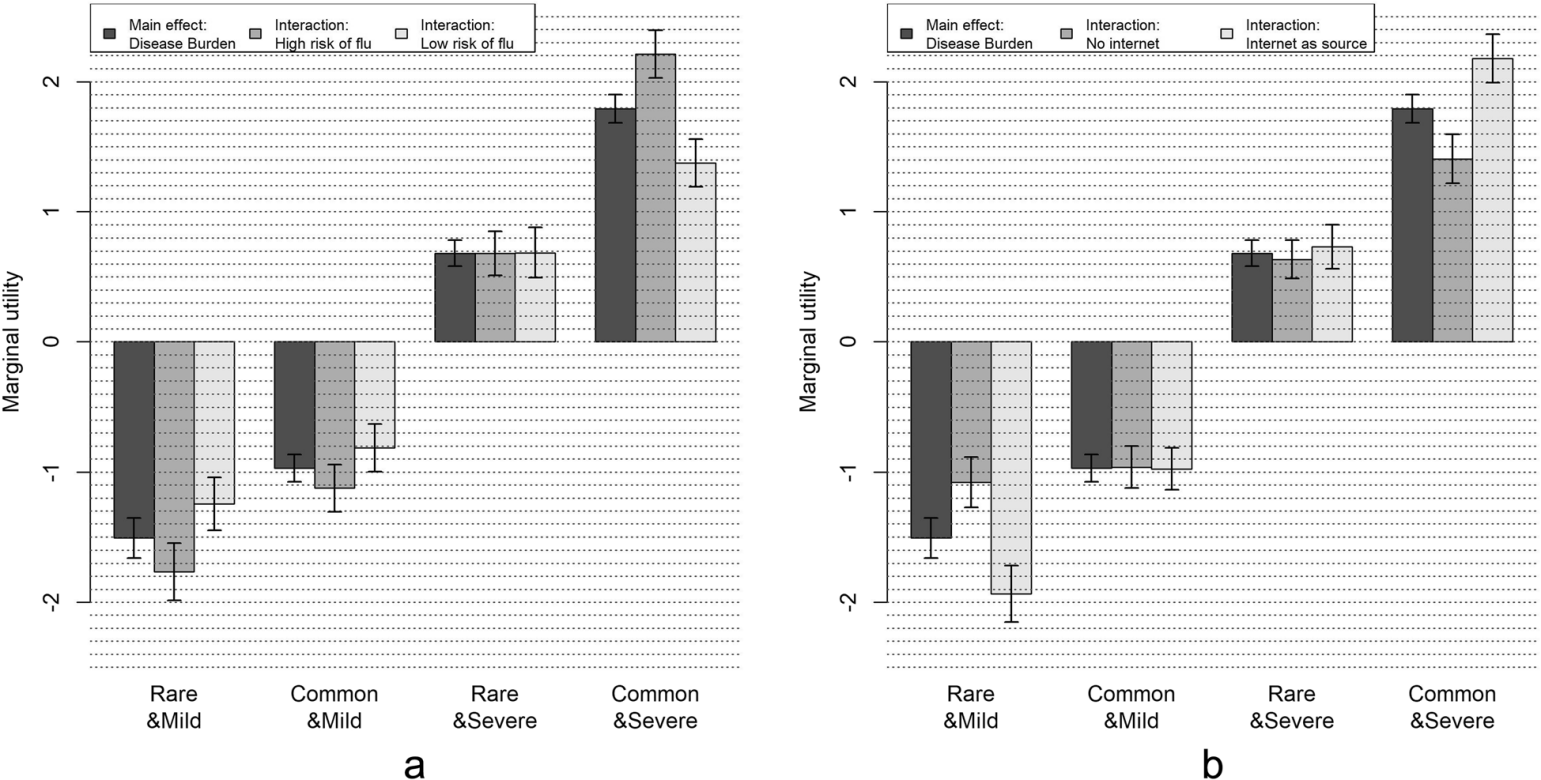
Marginal utilities for significant covariate interaction terms in the ‘child’ model. **a** Covariate interaction between burden of disease and risk of flu. **b** Covariate interaction between burden of disease and internet as source of information about vaccination (other than social media)

With regard to preference heterogeneity among the parents in the ‘child’ group (Fig. 3 and Table 5), we found significant interactions between burden of disease and the perceived susceptibility of their child to influenza and, additionally, between burden of disease and using information about vaccination from the internet (other than social media). Respondents who perceived the risk of their child to get influenza in the next 12 months as high were more in favor of a vaccine against a common and severe disease for their child, but also more in disfavor of a vaccine against a rare and mild disease, compared to those who considered their child to be less vulnerable to influenza. A similar pattern was found for the interaction with vaccine information from the internet. Respondents who consulted internet sources (36.6%) showed a stronger preference for vaccination against the common and severe level, while being more negative towards vaccinating against a rare and mild disease.

## Discussion

We identified the most important attributes for vaccination decision-making among Dutch adults, both for vaccinating themselves and for vaccinating their child. We found slight differences between these two perspectives. Although decision-making in both groups was primarily driven by vaccine effectiveness, burden of disease was more important for those deciding for their child, compared to those making a decision for themselves. The attribute burden of disease was expressed both in terms of disease severity (impact) and susceptibility (disease frequency). Of these two elements, more weight was assigned to severity, based on the attribute level utility estimates. Irrespective of perspective, the frequency of mild VRSE, population coverage and local coverage among friends and family were relatively unimportant in the decision. When adults decided for themselves, there was no significant difference in utility between vaccinating against a common or rare mild disease. In case of the decision for their child, a vaccine against a common and mild disease was preferred over a vaccine that protects against a mild but rare disease.

We found covariate interactions that differed between the two groups, whereby being among the target group for the annual influenza vaccination was the strongest in the ‘oneself’ model. Understandably, for respondents receiving this invitation, the question to vaccinate oneself was possibly related to different experiences compared to respondents not receiving the invitation, as this group is more experienced with vaccination overall compared to adults who do not qualify for influenza vaccination, and probably only have experience with travel vaccines for themselves. Following this logic, it is also not unexpected that this covariate is not explaining differences within the ‘child’ model. Notably, this also has to do with selection effects, because belonging to the risk group, thus receiving yearly influenza vaccination, is less likely in the group of parents belonging to the child group.

Contrary to what we expected, we were not able to find differences in preferences between respondents belonging to the orthodox Protestant community and other respondents. It is possible that those who opt out of vaccination may have chosen not to cooperate in this survey in the first place. Furthermore, moderately negative attitudes among respondents about vaccination in general (Table 3) may have been suppressed by the forced choice for either of two “pro-”vaccination options in the DCE section.

Attitudes towards vaccination are important determinants of vaccine acceptance and vaccine hesitancy [6, 8, 9, 11, 47]. We found a considerable number of neutral responses on almost all items in the attitudinal questionnaire, ranging from 12.6 to 48.2%. This might indicate that most participants are passively accepting routinely offered vaccinations. This could be linked to the highly reported trust in both information received from healthcare providers and government (> 70%). As described, all attitudinal items were used to assess preference heterogeneity and all, except for one in the ‘oneself’ model, showed no significant interactions in the joint models.

Of particular interest is the finding that the marginal utility estimates of the local (friends and family) and population coverage attributes were positive. This indicates that a vaccine is more likely to be accepted by an individual if others accept it too. A possible explanation is that individuals perceive a high coverage as a social norm or as public confidence in the vaccination programme. This link is also reported in Belgium [24], Australia [18], USA [19] and South Africa [23]. This observation is contrasting with the rational utility theory, which argues that when (enough) others are vaccinated you maximize your own utility by not vaccinating. Indeed, high coverages protect susceptible individuals via ‘herd protection’, without them being exposed to the (perceived) side-effects of vaccination [16, 17]. Furthermore, the observation that there is more willingness to vaccinate when acceptance at local or national level is high, is important in planning communication strategies about vaccination programmes. A message that the acceptance is high among those who have been offered vaccination recently may lead to a higher uptake among those who will be offered vaccination. Similarly, a message that the current coverage is low may lead to a lower uptake. Future studies should aim to quantify or further explain the relationship between reporting vaccination coverage and willingness to vaccinate. In the ‘child’ group, accessibility and mild VRSE significantly interacted with local coverage, making the effect of social influence even stronger. Both more common VRSE and vaccines that require co-payment were valued less negative if the local coverage was higher.

This study has several limitations. A first limitation is that although our sample was representative with respect to gender, age and province, there could be unmeasured differences between the respondents in our sample and the general population, for example due to higher computer access in the online panel. Moreover, the sample population in this study was higher educated compared to the population’s educational level.

A potential weakness regarding the DCE is the sensitivity to the framing of the VRSE attribute. In the original study in Flanders, this attribute appeared most important in both the vaccination decision for oneself and one’s child. In our DCE as well as in the South African DCE, this attribute was rephrased. Originally, the VRSE attribute did not provide any explanation with regard to severity of VRSE. Therefore, its description was changed such that it explicitly states that VRSE are of mild nature and rarely result in hospitalization. In our study, only the frequency of VRSE occurrence was varied. The differences in outcomes between our study and the South African study on the one hand, and the original Flemish study on the other, highlight the influence of framing, as well as being as specific as possible with regard to VRSE.

Another limitation is that due to the age structure of respondents with children under the age of 18, which are mostly between 25 and 40 years old, we observed a slight underrepresentation of this age group in the ‘oneself’ group. As a result, we cannot fully explore the differences between individuals with and without young children. However, given that we found only limited differences between the two vaccination decision-making settings and the large sample size, we do not expect that additional research would lead to different conclusions.

When the findings of this study are compared to both the Flemish and the South African study we find both similarities and differences. With regard to ranking of importance of the attributes the three studies all found different rank orders. As described, in the Flemish study VRSE combined with accessibility were the two most important attributes, followed by effectiveness and burden of disease. In South Africa, vaccine effectiveness was the most important attribute followed by population coverage and burden of disease. As in South Africa, here we found that vaccine effectiveness was the most important attribute, although in case of the child vaccination, this was combined with burden of disease. The importance of vaccine effectiveness was also found in other DCEs [20, 35, 38]. Per study, the interaction terms indicating preference heterogeneity differed slightly. In both this study and the Flemish study it was found that there was preference heterogeneity related to age and burden of disease. In the Flemish study it was found that the older age groups are less sensitive to burden of disease, i.e. the estimates between the different levels do not vary much. Here, this was the case with regard to belonging to the ‘influenza risk group’, which is related to age. None of the studies found considerable differences between the decision to vaccinate oneself or one’s child. Further, for all studies the estimates of the coverage attributes were positive, indicating that a higher coverage was preferred adding to evidence that decisions of peers are important. With regard to representation of the study population, all three studies struggled with underrepresentation of the groups with lower educational attainment.

## Conclusions

We performed a discrete choice experiment in The Netherlands to gain insights into vaccination decision-making with respect to vaccinating oneself and one’s child. Vaccine effectiveness, burden of disease and accessibility were the most important attributes in the decision-making process. Less influential, but still important contributors to vaccine utility were the population and local coverage. For both coverage attributes, we found positive utility estimates, indicating the effect of social influence. In the decision to vaccinate one’s child, estimates were even larger in absolute magnitude due to interactions with mild VRSE and accessibility, where higher local coverage increased the utility in magnitude for the least desirable options (i.e. common VRSE and co-payment). Our findings indicate that the focus of communication about vaccination is similar for vaccinating children and oneself. The message should stress the effectiveness and low effort of vaccination and clearly explain the burden of disease against which vaccines protect. Reporting high uptake rates may help to increase uptake of vaccination in future vaccination decisions.

## Supplementary information

**Additional file 1.** DCE Questionnaire

## Data Availability

The datasets used and/or analysed during the current study are available from the corresponding author on reasonable request.

## Abbreviations

DCE: Discrete choice experiment
LR: Likelihood Ratio
NIP: National Immunization Program
PML: Panel Mixed Logit
RI: Relative Importance
SBSQ: Set of Brief Screening Questions
VRSE: Vaccine-related side effects
