# Supplementary material for: Preferential differences in vaccination decision-making for oneself or one’s child in The Netherlands: a discrete choice experiment
Source: BMC Public Health. 2020 Jun 1;20:828. doi: 10.1186/s12889-020-08844-w (PMC7268356; doi:10.1186/s12889-020-08844-w)
Supplement: Supplementary file 1 — Additional file 1. DCE Questionnaire [file 12889_2020_8844_MOESM1_ESM.docx]

## Additional Material 1

### DCE Questionnaire

Q1: Your sex

- Female (1)
- Male (2)

Q2 Your age in years

*Check if number [18-85]*

Q3 Your 4-digit postal code

*Check if 4-digit number*

Q4 What is your highest completed education? *(Single answer)*

o Primary education (1)
o Lower vocational education (2)
o Preparatory secondary vocational education (3)
o Vocational education (4)
o (senior general secondary/university preparatory) education (5)
o University of Applied Sciences (6)
o University of Research (7)
o Other, namely: (8)

Q5 Which of the situations below best describes your current family situation *(Single answer)*

o Single (1)
o Single with children (2)
o Living together (married or not) without children (3)
o Living together (married or not) with children (4)
o None of the above (5)

If Q5= (2) or (4) show Q6

Q6 How many children do you have who are younger than 18? *(Single answer)*

o 0 (1)
o 1 (2)
o 2 (3)
o 3 (4)
o 3 + (5)

If Q6 is shown and if Q6≠ (1) show Q7

Q7 What is the birth year of your youngest child?

Q8 What is your mother's country of birth?

o The Netherlands (1)
o A different country within Western Europe (2)
o A different, not Western European country (3)

Q9 Do you work or have you ever worked in healthcare?

o No (1)
o Yes, what was your function? (2)
o Nurse (2.1)
o Doctor/physician (2.2)
o Paramedical profession (2.3)
o Pharmacist (2.4)
o Other (2.5)

Q10 Have you ever had a serious illness in yourself? (For example, cancer)

o Yes (1)
o No (2)

Q11 You will receive an invitation for the annual flu shot from your doctor?

o Yes (1)
o No (2)

If Q11 is 1, show Q12

Q12 Are you getting vaccinated with the annual flu shot?

o Never (1)
o Sometimes (2)
o Mostly (3)
o Always (4)

Q13 Do you or have you smoked?

O Yes, I smoke (1)
o I have smoked, but I quit (2)
o I have never smoked (3)

Q14 Does one of the beliefs below affect your thinking about vaccination? *If 1 answer is chosen, cannot pick option 5*

o Religious belief (1)
o Homeopathy (2)
o Natural medicines (3)
o Anthroposophy (4)
o None of the above (5)
o Others, namely: (6)

Q15 **To which faith or belief do you count yourself**?

O Protestant (1)
o Roman Catholic (2)
o Islamic (3)
o Jewish (4)
o Buddhism or Hinduism (5)
o Other faith or belief (6)
o I do not have a faith/belief (7)
o I wish not to answer this question (8)

Q16 To which specific Protestant denomination do you count?

o Protestant Church in the Netherlands (Dutch Reformed, Reformed, Lutheran), but not Reformed Bond (1)
o Reformed Union within the Protestant Church in the Netherlands (2)
o Restored Reformed Church (3)
o Reformed Municipalities (4)
o Reformed Municipalities in the Netherlands (5)
o Old Reformed Municipalities (6)
o Christian Reformed Churches (7)
o Reformed Churches (released) (8)
o Dutch Reformed Churches (9)
o Pentecostal churches and Gospel churches (10)
o Baptist Brotherhood (11)
o Remonstrant Brotherhood (12)
o Baptist churches (13)
o Other, namely____ (14)

If Q6 >1 show Q17B

Q17B Have you had your child (ren) vaccinated according to the National Vaccination Program? -

O Yes (1)
o No (2)
o Partly (3)
o Not applicable (4)

If Q17B == 1 or 3 show Q18B

Q18B What is your experience with vaccinating your children?

o Good (1)
o Bad (2)
o Not applicable (3)

*From now, until the end of the questionnaire, questions are either for group A, for group B or for all participants. Questions for all participants are displayed as Qx.
Group A consists of respondents who fill in the questionnaire for themselves. There are no explicit restricitons for this group. So, respondents that apply for group B too can, by randomisation, be
assigned to group A. Questions exclusively for group A are displayed as QxA
Groep B consists only of respondents with at least one child <18y Check by Q7. If Q7 is shown and ≠ (1), then assign persons to group B. This group fills in the questionnaire for decisions concerning
their youngest child. Questions exclusively for group B are displayed as QxB.*

Q19B A number of questions with statements about vaccination of children now follow. These statements concern **the** **decision to** **vaccinate your youngest child**. Would you like to choose the option that best describes your opinion for each statement?

| I think vaccinating my child according to the Dutch National Immunization Program is | Very unwise | Unwise - | Neutral | Wise - | Very wise |
| --- | --- | --- | --- | --- | --- |
| I think vaccinating my child according to the Dutch National Immunization Program is | Very unimportant | Unimportant | Neutral | Important | Very important |
| I think vaccinating my child according to the Dutch National Immunization Program is | Very unnecessary | Unnecessary | Neutral | Necessary | Very necessary |
| The people who are important to me think that I should have my child (ren) vaccinated | Strongly disagree | Disagree | Neutral | Agree | Strongly agree |
| The people who are important to me have their child(ren) vaccinated | Strongly disagree | Disagree | Neutral | Agree | Strongly agree |
| The people who are important to me appreciate it if I have my child(ren) vaccinated | Strongly disagree | Disagree | Neutral | Agree | Strongly agree |
| If I do not have my child vaccinated, there is a good chance that he / she will get an infectious disease that will be vaccinated within the National Vaccination Program | Strongly disagree | Disagree | Neutral | Agree | Strongly agree |
| The National Immunization Program is right for protecting the health of my child | Strongly disagree | Disagree | Neutral | Agree | Strongly agree |
| I think it is important that vaccinating my child contributes to the protection of others | Strongly disagree | Disagree | Neutral | Agree | Strongly agree |
| The diseases that are being vaccinated can be very serious | Strongly disagree | Disagree | Neutral | Agree | Strongly agree |
| The side effects of the vaccinations within the National Vaccination Program can be very serious | Strongly disagree | Disagree | Neutral | Agree | Strongly agree |
| Vaccinating my child is something I don't have to think about for long | Strongly disagree | Disagree | Neutral | Agree | Strongly agree |
| Vaccinating my child something obvious | Strongly disagree | Disagree | Neutral | Agree | Strongly agree |
| If I had to make the choice now, I would have my child vaccinated within the National Vaccination Program | Strongly disagree | Disagree | Neutral | Agree | Strongly agree |
| If I had to make an extra appointment with my health care provider (* health care provider is your GP or health center / pediatrician) for vaccination, that would be a reason not to have my child (ren) vaccinated | Strongly disagree | Disagree | Neutral | Agree | Strongly agree |
| I trust the information about vaccinations that my health care provider (* health care provider is your GP or health clinic / pediatrician) gives me | Strongly disagree | Disagree | Neutral | Agree | Strongly agree |
| I trust the information about vaccinations that I receive from the government | Strongly disagree | Disagree | Neutral | Agree | Strongly agree |
| I believe that it is the responsibility of every parent to have his / her child vaccinated within the National Vaccination Program | Strongly disagree | Disagree | Neutral | Agree | Strongly agree |
| I think it is bad if parents do not have their children vaccinated within the national vaccination program | Strongly disagree | Disagree | Neutral | Agree | Strongly agree |
| Experiencing infectious diseases contributes to a positive mental and physical development of my child. | Strongly disagree | Disagree | Neutral | Agree | Strongly agree |
| Experiencing infectious diseases leads to better and lifelong protection than a vaccination | Strongly disagree | Disagree | Neutral | Agree | Strongly agree |

Q19A A number of questions with vaccination statements now follow. The statements concern **the decision to vaccinate yourself**. Would you like to choose the option that best describes your opinion for each statement?

| I think getting myself vaccinated against infectious diseases | Very unwise | Unwise - | Neutral | Wise - | Very wise |
| --- | --- | --- | --- | --- | --- |
| I think getting myself vaccinated against infectious diseases | Very unimportant | Unimportant | Neutral | Important | Very important |
| I think getting myself vaccinated against infectious diseases | Very unnecessary | Unnecessary | Neutral | Necessary | Very necessary |
| The people who are important to me think I should get vaccinated | Strongly disagree | Disagree | Neutral | Agree | Strongly agree |
| The people who are important to me have themselves vaccinated | Strongly disagree | Disagree | Neutral | Agree | Strongly agree |
| The people who are important to me appreciate it if I have myself vaccinated | Strongly disagree | Disagree | Neutral | Agree | Strongly agree |
| If I don't get myself vaccinated, there is a good chance that I will get an infectious disease against which I have been vaccinated | Strongly disagree | Disagree | Neutral | Agree | Strongly agree |
| The vaccinations that are offered are good for the protection of my health | Strongly disagree | Disagree | Neutral | Agree | Strongly agree |
| I think it is important that vaccinating myself contributes to the protection of others | Strongly disagree | Disagree | Neutral | Agree | Strongly agree |
| The diseases that are being vaccinated can be very serious | Strongly disagree | Disagree | Neutral | Agree | Strongly agree |
| The side effects of the vaccinations within the National Vaccination Program can be very serious | Strongly disagree | Disagree | Neutral | Agree | Strongly agree |
| Vaccinating myself is something I don't have to think about for long | Strongly disagree | Disagree | Neutral | Agree | Strongly agree |
| Vaccinating myself is a matter of course | Strongly disagree | Disagree | Neutral | Agree | Strongly agree |
| If I had to make the choice now, I would have myself vaccinated | Strongly disagree | Disagree | Neutral | Agree | Strongly agree |
| If I had to make an extra appointment with my health care provider (* health care provider is your general practitioner or health clinic) for vaccination, that would be a reason not to have my vaccine vaccinated | Strongly disagree | Disagree | Neutral | Agree | Strongly agree |
| I have faith in the information about vaccinations that my health care provider (* health care provider is your general practitioner or health clinic ) gives me | Strongly disagree | Disagree | Neutral | Agree | Strongly agree |
| I have faith in the information about vaccinations that I receive from the government | Strongly disagree | Disagree | Neutral | Agree | Strongly agree |
| I think it is everyone's responsibility to vaccinate | Strongly disagree | Disagree | Neutral | Agree | Strongly agree |
| I find it bad if people do not get vaccinated within the national vaccination program. | Strongly disagree | Disagree | Neutral | Agree | Strongly agree |
| Experiencing infectious diseases contributes to a positive mental and physical development | Strongly disagree | Disagree | Neutral | Agree | Strongly agree |
| Experiencing infectious diseases leads to better and lifelong protection than a vaccination | Strongly disagree | Disagree | Neutral | Agree | Strongly agree |

Q20B Who are important to you if you have to decide whether or not to have your child vaccinated? (Multiple answers possible) -

o Partner (1)
o Family (2)
o Friends (3)
o Other parents (4)
o Colleague’s (5)
o General practitioner (6)
o Youth healthcare centre/Public Health Service (7)
o Others, namely: (8)

Q20A Who are important to you if you have to decide whether or not to have yourself vaccinated? (Multiple answers possible)

o Partner (1)
o Family (2)
o Friends (3)
o Other parents (4)
o Colleague’s (5)
o General practitioner (6)
o Youth healthcare centre/Public Health Service (7)
o Others, namely: (8)

Q21 The following questions describe two situations where you can make a choice.

Q22A Below we provide a simplified example. We always ask in which of the two situations you are **most likely to have a vaccine administered to you**. The two choices you can make differ on some characteristics.

| 30% of the Dutch population has already been vaccinated | 30% of the Dutch population has already been vaccinated |
| --- | --- |
| Slight side effects are rare and serious side effects are very rare | Minor side effects are common and serious side effects are very exceptional |
| o | o |

Q22B Below we provide a simplified example. We always ask in which of the two situations are the **most likely to have your child administer a vaccine**. The two choices you can make differ on some characteristics.

| 30% of the Dutch population has already been vaccinated | 30% of the Dutch population has already been vaccinated |
| --- | --- |
| Slight side effects are rare and serious side effects are very rare | Minor side effects are common and serious side effects are very exceptional |
| o | o |

Q23 --- *10 choice sets from conjoint design: excel file ---*

Q24A How seriously would you estimate the impact on your health if you have one of the following diseases get in the next year?

| **Seriousness/severity of disease** | Very serious  (1) | Serious - (2) | Moderately serious (3) | Not serious (4) | Not serious at all (5) |
| --- | --- | --- | --- | --- | --- |
| Flu/influenza (1) | o | o | o | o | o |
| Leukaemia (2) | o | o | o | o | o |
| Bladder infection (3) | o | o | o | o | o |
| Measles (4) | o | o | o | o | o |

Q24B How seriously would you estimate the impact on the health of your youngest child if your child has one of the following diseases in the next year? -

| **Seriousness/severity of disease** | Very serious (1) | Serious (2) | Moderately serious (3) | Not serious (4) | Not serious at all (5) |
| --- | --- | --- | --- | --- | --- |
| Flu/influenza (1) | o | o | o | o | o |
| Leukaemia (2) | o | o | o | o | o |
| Bladder infection (3) | o | o | o | o | o |
| Measles (4) | o | o | o | o | o |

Q25A How high do you estimate the chance that you will get one of the following diseases during the next 12 months.

| **Risk of diseases** | Very high chance (1) | High chance (2) | Average chance (3) | Small chance (4) | Very small chance (5) |
| --- | --- | --- | --- | --- | --- |
| Flu/influenza (1) | o | o | o | o | o |
| Leukaemia (2) | o | o | o | o | o |
| Bladder infection (3) | o | o | o | o | o |
| Measles (4) | o | o | o | o | o |

Q25B How high do you estimate the chance that your youngest child will get one of the following diseases during the next 12 months.

| **Risk of diseases** | Very high chance (1) | High chance (2) | Average chance (3) | Small chance (4) | Very small chance (5) |
| --- | --- | --- | --- | --- | --- |
| Flu/influenza (1) | o | o | o | o | o |
| Leukaemia (2) | o | o | o | o | o |
| Bladder infection (3) | o | o | o | o | o |
| Measles (4) | o | o | o | o | o |

Q26 How do you get or where do you look for information about infectious diseases and vaccination?

o on social media (Facebook, twitter etc.) (1)
o via traditional media (newspaper, television or printed magazines (2)
o via the official website of the National Institute for Public Health and the Environment or the National immunization program (3)
o via internet (other than social media (4)
o friend and/or family (5)
o via my general practitioner, child care physician or paediatrician (6)
o None of the above (7)

People often find the information they receive about their health or illness unclear or complicated. We are curious about your experiences with this

Q28 How often does someone help you to read letters and leaflets from your general practitioner or the hospital?

o Never (1)
o Now and then (2)
o Sometimes (3)
o Often (4)
o Always (5)

Q29 How certain are you that you fill in medical forms correctly?

o Very much (1)
o Fairly (2)
o A bit (3)
o A little bit (4)
o Not at all (5)

Q30 How often is it difficult for you to learn more about your health because you do not understand written information?

o Never (1)
o Now and then (2)
o Sometimes (3)
o Often (4)
o Always (5)

We thank you for participating to our survey
